# Brorin is required for neurogenesis, gliogenesis, and commissural axon guidance in the zebrafish forebrain

**DOI:** 10.1371/journal.pone.0176036

**Published:** 2017-04-27

**Authors:** Ayumi Miyake, Yoko Mekata, Hidenori Fujibayashi, Kazuya Nakanishi, Morichika Konishi, Nobuyuki Itoh

**Affiliations:** Department of Genetic Biochemistry, Graduate School of Pharmaceutical Sciences, Kyoto University, Sakyo, Kyoto Japan; Centre National de la Recherche Scientifique, FRANCE

## Abstract

Bmps regulate numerous neural functions with their regulators. We previously identified Brorin, a neural-specific secreted antagonist of Bmp signaling, in humans, mice, and zebrafish. Mouse Brorin has two cysteine-rich domains containing 10 cysteine residues in its core region, and these are located in similar positions to those in the cysteine-rich domains of Chordin family members, which are secreted Bmp antagonists. Zebrafish Brorin had two cysteine-rich domains with high similarity to those of mouse Brorin. We herein examined zebrafish *brorin* in order to elucidate its *in vivo* actions. Zebrafish *brorin* was predominantly expressed in developing neural tissues. The overexpression of *brorin* led to the inactivation of Bmp signaling. On the other hand, the knockdown of *brorin* resulted in the activation of Bmp signaling and *brorin* morphants exhibited defective development of the ventral domain in the forebrain. Furthermore, the knockdown of *brorin* inhibited the generation of γ–aminobutyric acid (GABA)ergic interneurons and oligodendrocytes and promoted the generation of astrocytes in the forebrain. In addition, *brorin* was required for axon guidance in the forebrain. The present results suggest that Brorin is a secreted Bmp antagonist predominantly expressed in developing neural tissues and that it plays multiple roles in the development of the zebrafish forebrain.

## Introduction

During embryonic development of the vertebrate brain, the neural plate undergoes regional subdivisions into the forebrain, midbrain, hindbrain, and spinal cord. The forebrain is subdivided into the secondary prosencephalon, consisting of the telencephalon and hypothalamus, and the diencephalon undergoes subdivisions into the thalamus, prethalamus, zona limitans intrathalamica (ZLI), and pretectum [1,2]. The telencephalon is also subdivided into the ventrally positioned subpallial telencephalon and dorsally located pallial telencephalon. Interactions between secreted signaling molecules are crucial for the regionalization and control of cell proliferation and also for the specification of cell fates in the telencephalic and diencephalic subdivisions. Bone morphogenetic proteins (Bmps) have numerous roles in neural development and Bmp signaling is involved in growth and patterning in the dorsal telencephalon [3–7]. In addition, the cross-regulation of Bmp, Wnt, and Fgf signaling is required for dorsal telencephalic patterning [5,6]. Bmps are also known to transform the fate of neural precursors from neurogenesis or oligodendrogliogenesis to astrogliogenesis [8–10]. In the ventral region of the forebrain, patterning is coordinated via Hedgehog (Hh) signaling, which is critical for specifying ventral forebrain neurons [11–13].

Bmps are secreted signaling molecules that are members of the TGF-β superfamily [14], and are subjected to regulation by numerous secreted regulators, including Noggin, Follistatin, FSRP, and members of the Chordin family and DAN/Cerberus family [15]. Brorin [also known as von Willebrand factor C domain-containing protein 2 (Vwc2)] was previously identified in mice, zebrafish, and humans [16,17]. Mouse Brorin has two cysteine-rich domains, the cysteines in which are located at similar positions to those in the domains of Chordin family members [16,18]. Brorin has been shown to inhibit the activity of Bmps *in vitro* [16]. In the mouse, *Brorin* is predominantly expressed in the neural tissues of embryos and adult animals, and Brorin reportedly promoted neurogenesis, but not astrogliogenesis, in cultured mouse neural precursor cells [16]. However, the role of Brorin in early neural development has not yet been elucidated.

We previously identified zebrafish *brorin* [17]. In the present study, we investigated zebrafish *brorin* activity during the embryonic development of the brain. We demonstrated that *brorin* inhibited Bmp signaling and played a critical role in the development of the ventral domain and specification of γ–aminobutyric acid (GABA)ergic interneurons and oligodendrocyte progenitors in the forebrain. It was also implicated in the suppression of astrocyte generation in the forebrain. Our results indicate that *brorin* is essential for the appropriate expression of axon guidance molecules and has a role in the formation of forebrain commissural axons.

## Materials and methods

### Husbandry

Zebrafish (*Danio rerio*) were maintained, embryos were obtained by natural spawning and cultured, and their developmental stages were evaluated as described previously [19]. These experiments were approved by and conducted according to the guidelines of the Institutional Animal Care and Use Committee of Kyoto University Graduate School of Pharmaceutical Sciences (protocol approval number: 2015–26).

### Reverse transcription-polymerase chain reaction (RT-PCR)

Expression profiles were assessed over time by RT-PCR using a pair of primers for an 806-bp fragment of *brorin* (5’-CTCTTGTACACAAGTGCACG-3’/ 5’-TAGCAGATGGTGCATTCGTC-3’) and zebrafish *elongation factor 1-α* (*ef1α*) [20].

### Whole mount *in situ* hybridization

Whole mount *in situ* hybridization was performed as described previously using digoxigenin-labeled RNA probes [21]. The *brorin* probe was synthesized using a plasmid containing full-length cDNA. The following probes were also employed: zebrafish *emx1* [22], *tbr1* [23], *dlx2a* [24], *shh* [25], *ngn1* [26], *ascl1a* [27], *gad1* [28], *plp* [29], *glula* [30], *netrin 1a* [31], and *sema3d* [32].

### Injection of RNA

The entire coding region of zebrafish *brorin* cDNA was inserted into a vector (pCS2+) [33]. Using a mMESSAGE mMACHINE kit (Ambion), capped *brorin* mRNA was synthesized from linearized *brorin* cDNA derived from pCS2+. mRNA was then diluted with water to 0.4 μg/μl and 1 nl was injected into 2-cell to 4-cell zebrafish embryos.

### Injection of morpholino oligonucleotides

After synthesis by Gene-Tools, LLC (Corvallis, OR), morpholino oligonucleotides (MOs) were diluted in Danieau buffer [34]. The two MOs used were splice site-targeted *brorin* MO1, with a 25-base antisense sequence corresponding to that between intron 1 and exon 2 of the coding region (5’-ATGGAGACACCTAGAAGAACAAACC-3’), and splice site-targeted *brorin* MO2, with a 25-base antisense sequence corresponding to that between exon 1 and intron 1 of the coding region (5’-CACTTAATGTGCTGCTCTAACCTTA-3’). The control MO sequence was 5’-CCTCTTACCTCAGTTACAATTTATA-3’ [35–37]. Either *brorin* MO1 (6 ng), *brorin* MO2 (12 ng), or control MO (12 ng) was injected into 2-cell to 4-cell zebrafish embryos.

In order to investigate the effectiveness of these MOs, RNA was isolated from embryos injected with control MO, *brorin* MO1, or *brorin* MO2, and RT-PCR was performed using the above primers.

### Immunohistochemistry

Whole mount immunostaining was performed as described previously [20] using rabbit anti-phospho-Smad1/5/8 (Cell Signaling) diluted to 1:100 [38,39] and mouse anti-acetylated tubulin (Sigma) diluted to 1:200 [40]. Alexa Fluor 488 goat anti-rabbit IgG (1:200; Invitrogen) or anti-mouse IgG (1:500; Invitrogen) was employed for the detection of fluorescence.

## Results

### Characterization of zebrafish *brorin*

**Fig 1:**
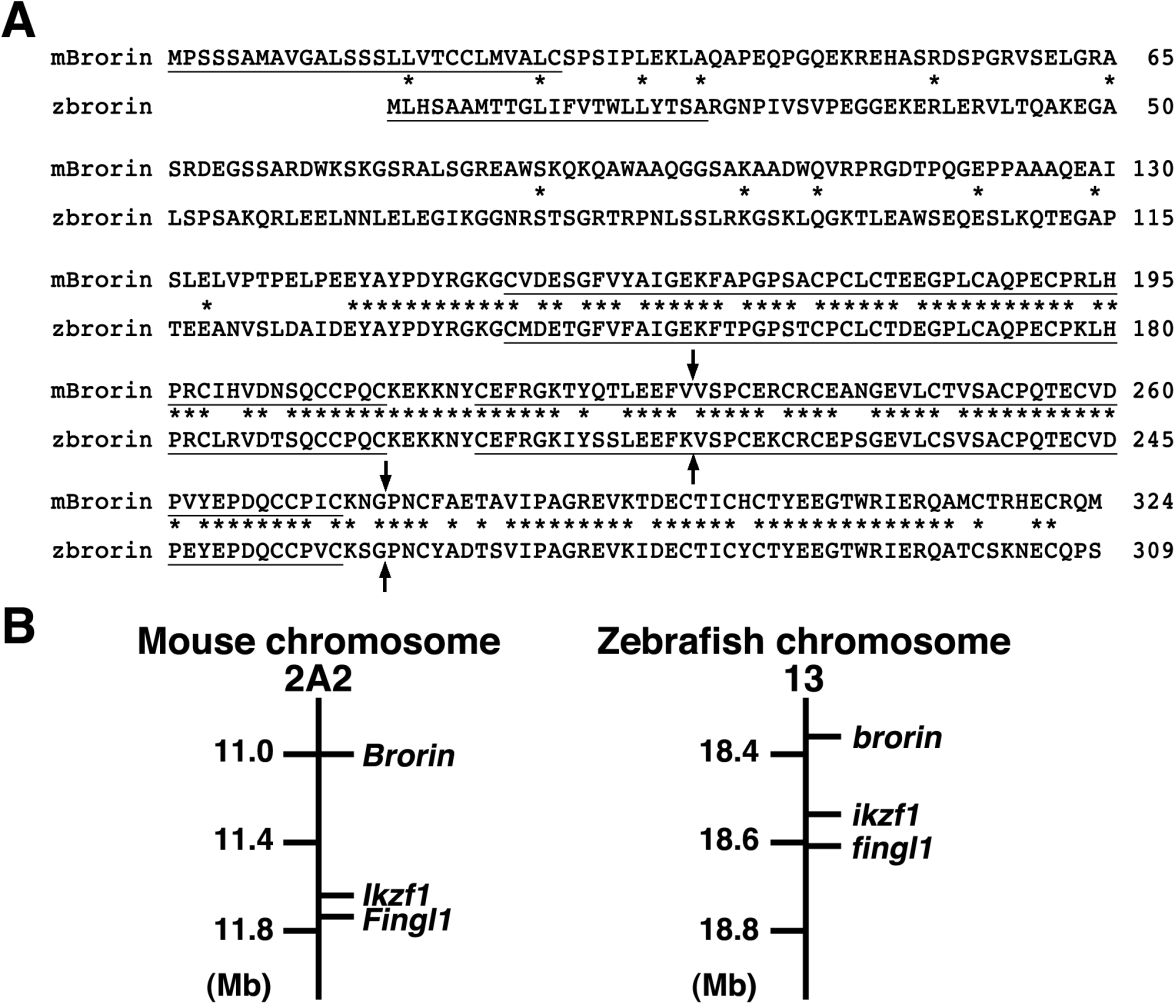
Molecular analysis of zebrafish Brorin. (A) Comparison of the amino acid sequences of mouse Brorin and zebrafish Brorin. The numbers refer to the amino acid positions of mouse and zebrafish Brorin. Asterisks and arrows indicate identical amino acid residues in the sequences and positions of introns, respectively. Underlining at the amino terminus and core sequence indicates the putative secreted signal sequence and cysteine-rich domains, respectively. (B) The syntenic relationship between mouse chromosome 2A2 and zebrafish chromosome 13. Mb, megabase.

We previously identified the zebrafish *brorin* gene in a homology-based search of zebrafish nucleotide sequences in GenBank using the amino acid sequence of mouse Brorin [17]. Zebrafish Brorin is presumed to be a secreted protein composed of 309 amino acids with a putative 22-amino acid signaling sequence at its amino-terminus (Fig 1A). In its core region, it has two cysteine-rich domains with high similarity to those of mouse Brorin (Fig 1A). While the 127-amino acid sequence of the amino-terminal region shares less similarity with that of mouse Brorin, the other regions of zebrafish Brorin and mouse Brorin are highly similar (~82% identity) (Fig 1A) [16].

The coding region of zebrafish *brorin* is divided into 2 introns. The coding region of mouse *Brorin* is also divided into 2 introns, with similar positions to those of zebrafish *brorin* (Fig 1A) [16]. Zebrafish *brorin* is closely linked to the *ikzf1* and *fingl1* genes on chromosome 13, while mouse *Brorin* is closely linked to the *Ikzf1* and *Fingl1* genes at A2 on chromosome 2 (Fig 1B). These results also indicate that zebrafish *brorin* is a zebrafish ortholog of mouse *Brorin*.

### Pattern of *brorin* expression in the brain

**Fig 2:**
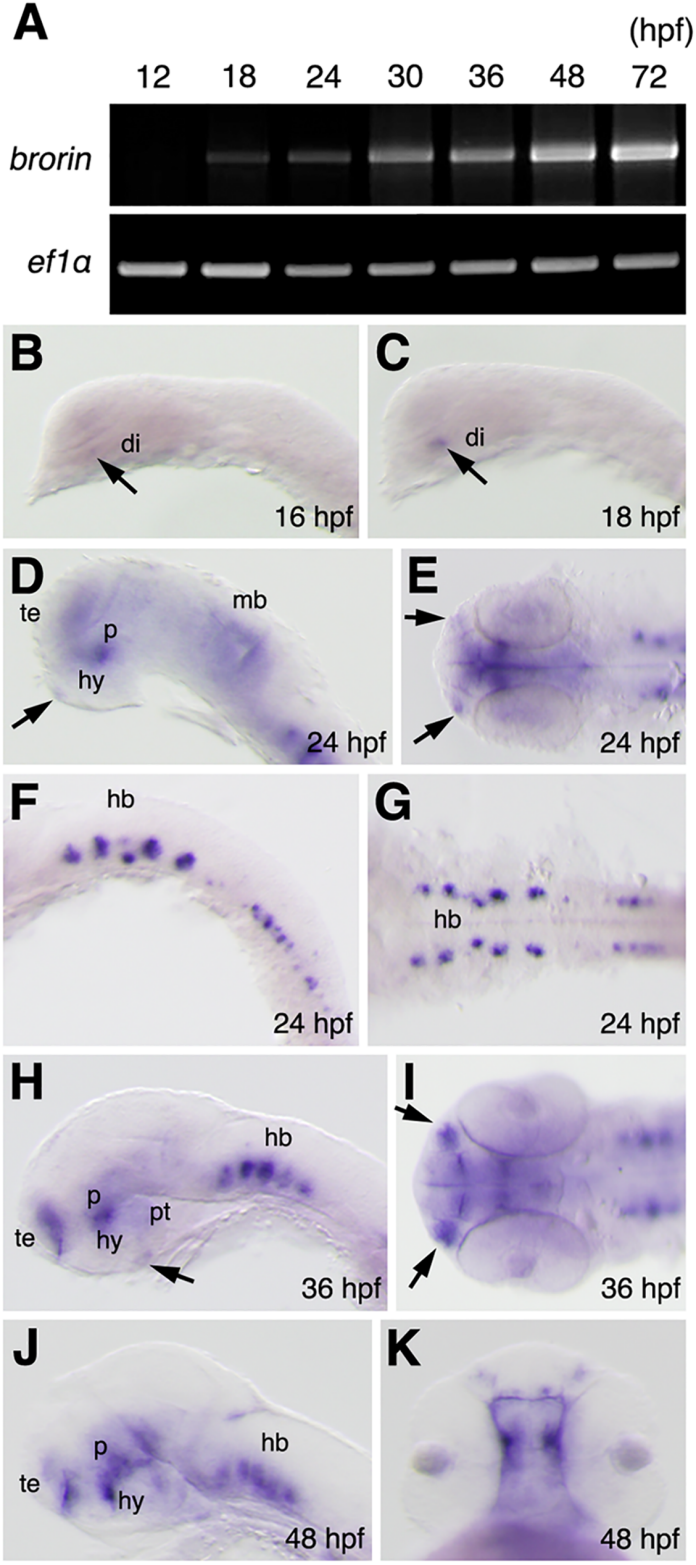
Pattern of *brorin* expression in zebrafish embryos. (A) Amplification of *brorin* by RT-PCR at the indicated stages (the lower panel shows *ef1α* as a control). (B-K) Expression of *brorin* in zebrafish embryos at the indicated stages as detected by whole-mount *in situ* hybridization. B-D, F, H, and J are lateral views, anterior to the left; E is the ventral view; G and I are dorsal views; K is the frontal view. Arrows in panels D and H indicate the pituitary gland. Arrows in panels E and I indicate the olfactory placode. ac, anterior commissure; di, diencephalon primordium; hb, hindbrain; hy, hypothalamus; mb, midbrain; p, prethalamus; pt, posterior tuberculum; te, telencephalon.

The expression of *brorin* in the brains of zebrafish embryos has already been reported at 36 hours post fertilization (hpf) [17]; however, its expression has not yet been examined at different stages of embryonic development. The time course of *brorin* expression during embryonic development was initially examined using RT-PCR. A RT-PCR analysis was performed using samples ranging between 3hpf and 3dpf. A low level of *brorin* expression was initially detected at 18 hpf, after which its expression gradually increased and was detected until at least 72 hpf (Fig 2A).

The spatiotemporal pattern of *brorin* expression in the embryonic zebrafish brain was subsequently investigated by whole mount *in situ* hybridization. A low level of *brorin* expression was initially observed in the diencephalon primordium at 16 hpf (Fig 2B). Its expression was also detected in the diencephalon primordium at 18 hpf (Fig 2C). At 24 hpf, *brorin* expression was detected in the telencephalon and prethalamic/alar hypothalamic region, (Fig 2D), as well as in several patches of cells in the hindbrain and spinal cord (Fig 2F and 2G). A low level of *brorin* expression was also noted in the posterior part of the midbrain, olfactory placode, and pituitary gland at 24 hpf (Fig 2D and 2E). At 36 hpf, *brorin* expression was detected in the ventral telencephalon, prethalamic/alar hypothalamic region, olfactory placode, hindbrain, and spinal cord (Fig 2H and 2I and data not shown). A low level of *brorin* expression was also found in the posterior tubercular and pituitary gland at 36 hpf (Fig 2H). This expression of *brorin* at 36 hpf was consistent with our previous findings [17]. Its expression in the forebrain, hindbrain, and spinal cord persisted until at least 48 hpf (Fig 2J and 2K and data not shown). Although the strong expression of *brorin* was still noted in the brain, greatly diminished in the olfactory placode (Fig 2K).

### Inhibition of *brorin* functions in zebrafish embryos

**Fig 3:**
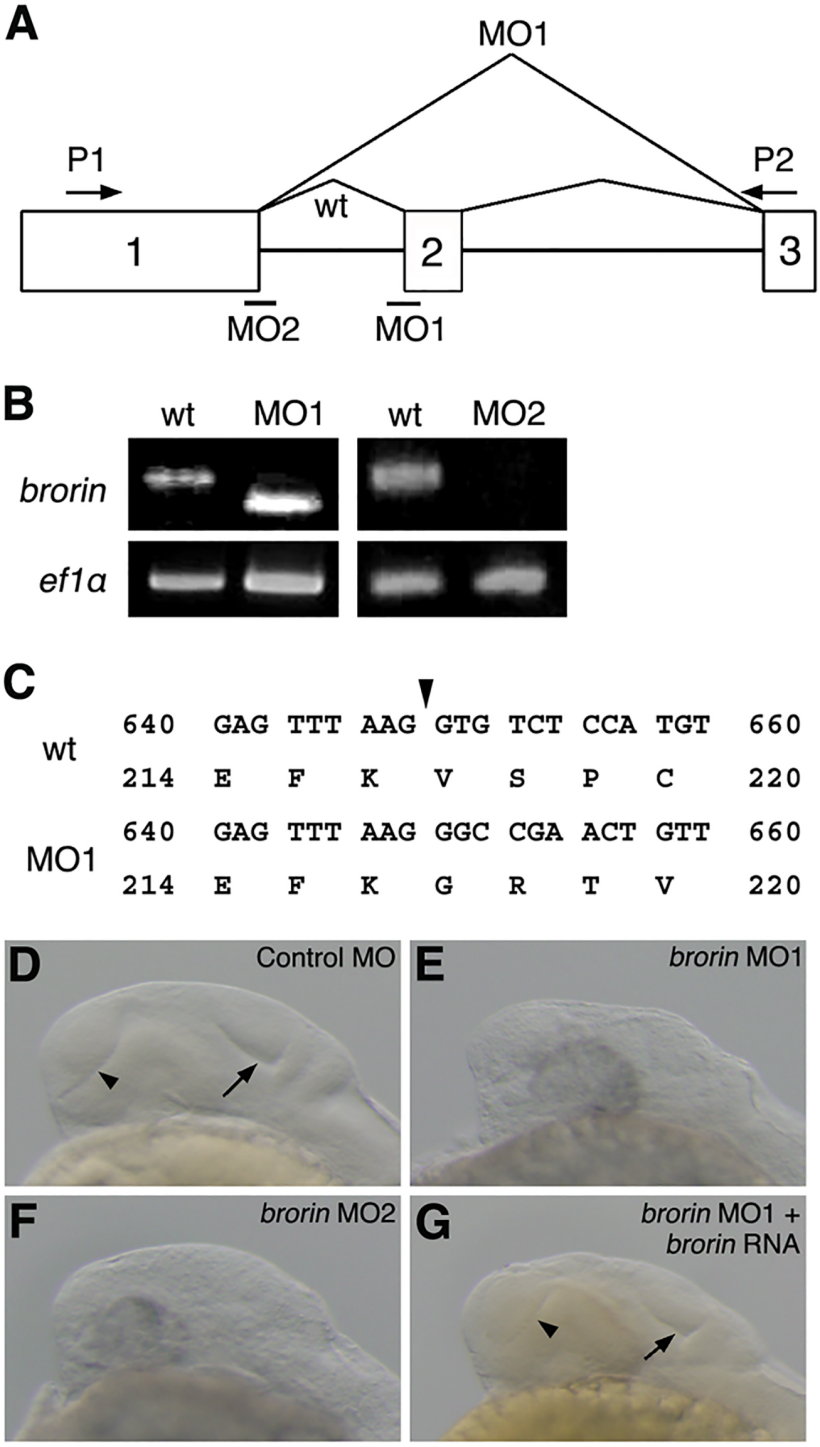
Inhibition of *brorin* function in zebrafish embryos. (A) The coding region of *brorin* is divided into two introns, with open boxes and black lines indicating exons and introns, respectively. MO indicates the target position of *brorin* MO. (B) *brorin* cDNA was amplified from the cDNA of wild-type embryos or *brorin* MO-injected embryos by RT-PCR using the P1 and P2 primers, the positions of which are indicated by arrows (A). (C) The nucleotide sequences of *brorin* cDNAs were elucidated. Numbers show the nucleotide sequence of the coding region and amino acid sequence, and arrowheads indicate splice sites between exons one and two. (D-G) Lateral views of control MO-injected (D), *brorin* MO1-injected (E), *brorin* MO2-injected (F), and *brorin* MO1- and *brorin* RNA-injected (G) embryos at 24 hpf. Arrows and arrowheads indicate the tectal ventricle and telencephalic ventricle, respectively. di, diencephalon; mb, midbrain; te, telencephalon.

In order to assess the role of *brorin* during the development of zebrafish, knockdown experiments were performed with MOs. Two different splice site-targeted MOs (MO1 and MO2) for *brorin* were injected into 2-cell embryos to examine whether splicing of the *brorin* mRNA precursor was efficiently blocked (Fig 3A). Amplified cDNA from *brorin* MO1-injected embryos was shorter than wild-type cDNA and underwent abnormal splicing to yield a truncated translation product (Fig 3A–3C). The expression of mature *brorin* mRNA was markedly decreased in embryos injected with *brorin* MO2 (Fig 3B). New bands at higher or lower molecular weights, which are indicative of cryptic splicing products or exon skipping, were not detected, suggesting the degradation of incorrectly spliced transcripts by nonsense-mediated decay. These results indicate that the two non-overlapping MOs both effectively blocked the maturation of *brorin* mRNA.

Embryos injected with control MO developed normally, whereas *brorin* morphants were morphologically defective in the formation of the boundary between the telencephalon and diencephalon, and tectal ventricle at 24 hpf (MO1, *n* = 175/185 and MO2, *n* = 29/29) (Fig 3D–3F). Furthermore, we investigated whether *brorin* RNA rescues the phenotype of *brorin* MO-injected embryos. We found that the co-injection of *brorin* RNA with *brorin* MO1 prevented the development of brain defects caused by *brorin* MO1 (*n* = 11/12) (Fig 3G). Thus, these results suggest that *brorin* is required for normal brain development.

### Effects of *brorin* on Bmp signaling

**Fig 4:**
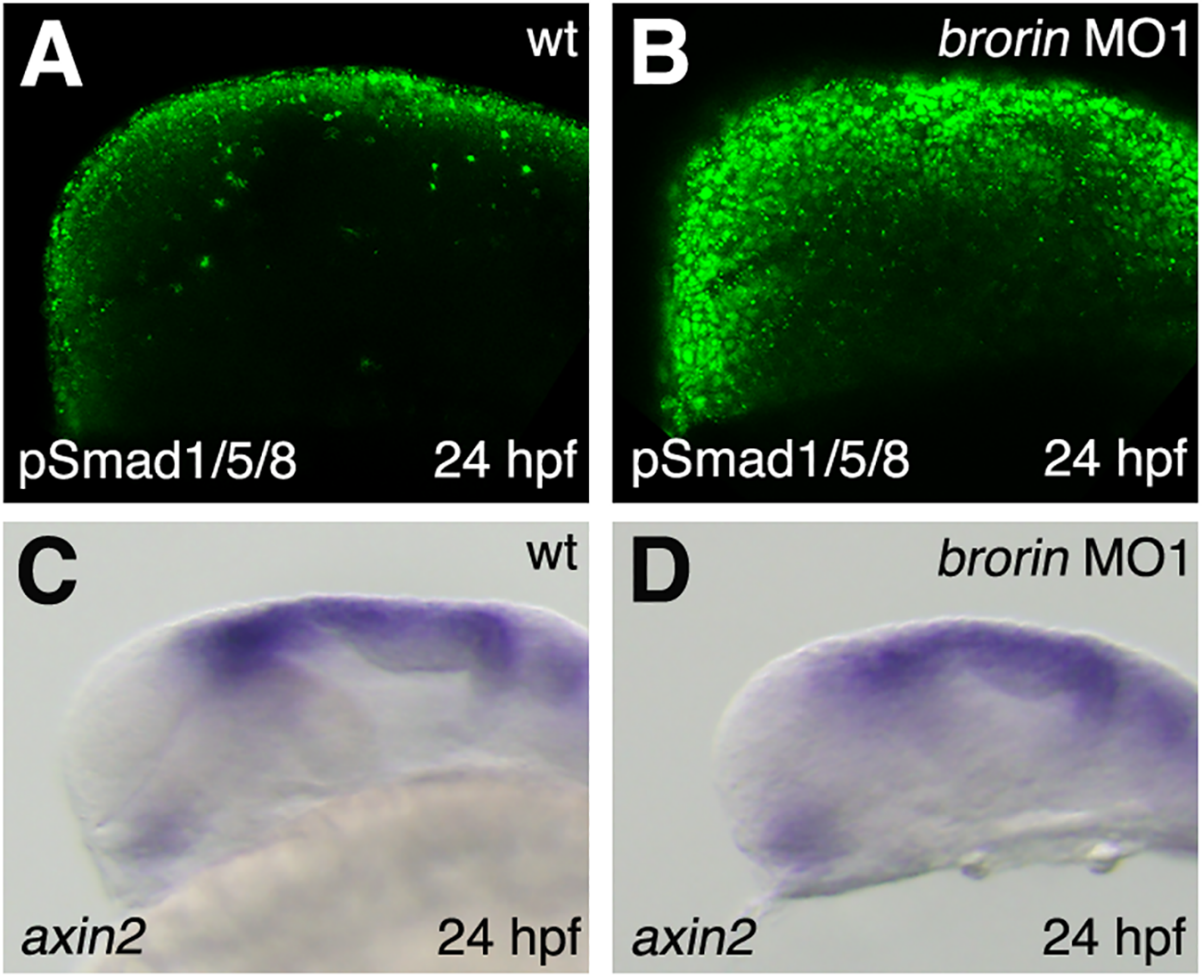
pSmad distribution and expression of the Wnt target gene in *brorin* morphants. (A, B) Pattern of pSmad expression in wild-type embryos (A) and *brorin* morphants (B) at 24 hpf. (C, D) Expression of *axin2* in wild-type embryos (C) and *brorin* morphants (D) at 24 hpf. Lateral view with the anterior to the left.

Since mouse Brorin has been shown to antagonize Bmp signaling *in vitro* [16], we investigated the effects of *brorin* knockdown on the Bmp signaling pathway in order to elucidate the mechanisms underlying the phenotypes of *brorin* morphants. The binding of Bmps to their receptors induces the phosphorylation of Smad proteins, after which phosphorylated Smad (pSmad) is translocated into the nucleus to regulate the transcription of various target genes [41]. Therefore, we examined the phosphorylation of Smad proteins using an antibody that recognizes phosphorylated Smads 1, 5, and 8 in embryos injected with *brorin* MO at 24 hpf. At 24 hpf, pSmad was detected in dorsal cells in the brains of wild-type embryos (Fig 4A). In *brorin* morphants, pSmad was increased in the dorsal region of the brain (MO1, *n* = 11/11 and MO2, *n* = 18/18) (Fig 4B, and data not shown). This result indicates that the inhibition of *brorin* leads to the activation of Bmp signaling.

**Fig 5:**
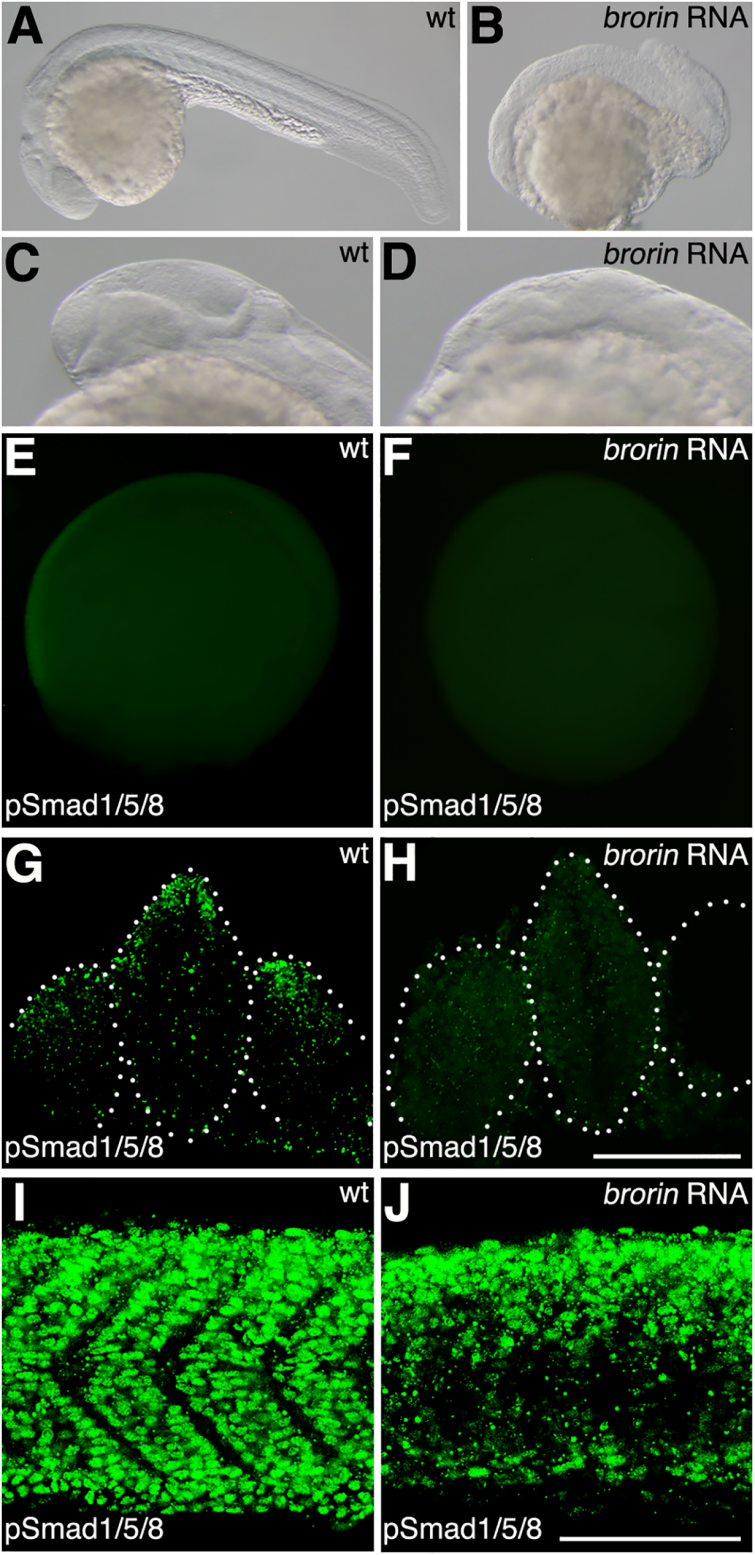
pSmad distribution in *brorin* RNA-injected embryos. (A-D) Lateral views of wild-type (A, C) and *brorin* RNA-injected (B, D) embryos at 24 hpf. (E, F) Pattern of pSmad expression in wild-type (E) and *brorin* RNA-injected (F) embryos at 8 hpf. (G-J) Pattern of pSmad expression in wild-type (G, I) and *brorin* RNA-injected (H, J) embryos at 24 hpf. G and H are optical cross-sections; I and J are lateral views anterior to the left. Scale bar: 50 μm.

In order to establish whether Brorin inhibits the Bmp signaling pathway *in vivo*, we performed gain-of-function experiments. In early zebrafish embryos, Bmps are expressed in the ventral margin of the blastula and a ventral-to-dorsal gradient of Bmp activity is essential for patterning of the dorsoventral axis. The non-axial region of the tail is lost in the zebrafish mutants *bmp2b*/*swirl*, *bmp7*/*snailhouse*, and *smad5/somitabun* [42–45]. Similar phenotypes have been observed after the misexpression of a Bmp inhibitor such as *noggin1* [46]. Accordingly, the inhibition of Bmp signaling prevents tail development. At 24 hpf, embryos injected with *brorin* RNA exhibited morphological abnormalities in the brain and defects in the tail (*n* = 39/43) (Fig 5A–5D). In order to investigate the effects of *brorin* overexpression on the Bmp signaling pathway, we examined the phosphorylation of Smad1/5/8 in embryos injected with *brorin* RNA at 8 and 24 hpf. Consistent with the above results, pSmad 1/5/8 staining was not detected in the ventrolateral domain of embryos injected with *brorin* RNA at 8 hpf (*n* = 22/25) (Fig 5E and 5F). At 24 hpf, pSmad was detected in the dorsal cells of the forebrain and eye in wild-type embryos, but not in embryos injected with *brorin* RNA (*n* = 11/11) (Fig 5G and 5H). Furthermore, a decrease in pSmad in the ventral part of the somite was observed in *brorin* RNA-injected embryos that exhibited a mild defect in the tail, whereas high levels of pSmad were detected in wild-type embryos (*n* = 19/22) (Fig 5I and 5J). These results indicate that the overexpression of *brorin* leads to the inactivation of Bmp signaling.

Dorsalization is also caused by the inhibition of the Wnt signaling pathway and embryos lacking *wnt8* display a similar phenotype to that caused by the inhibition of the Bmp signaling pathway [46]. Therefore, in order to investigate the effects of *brorin* knockdown on the Wnt signaling pathway, we examined the expression of *axin2*, which is a direct target gene of the canonical Wnt signaling pathway, in *brorin* morphants at 24 hpf. In *brorin* morphants, the expression of *axin2* was not increased in the brain (MO1, *n* = 13/13) (Fig 4C and 4D). These results indicate that Brorin inhibits Bmp signaling, but not canonical Wnt signaling.

### Effects of *brorin* knockdown on patterning in the forebrain

**Fig 6:**
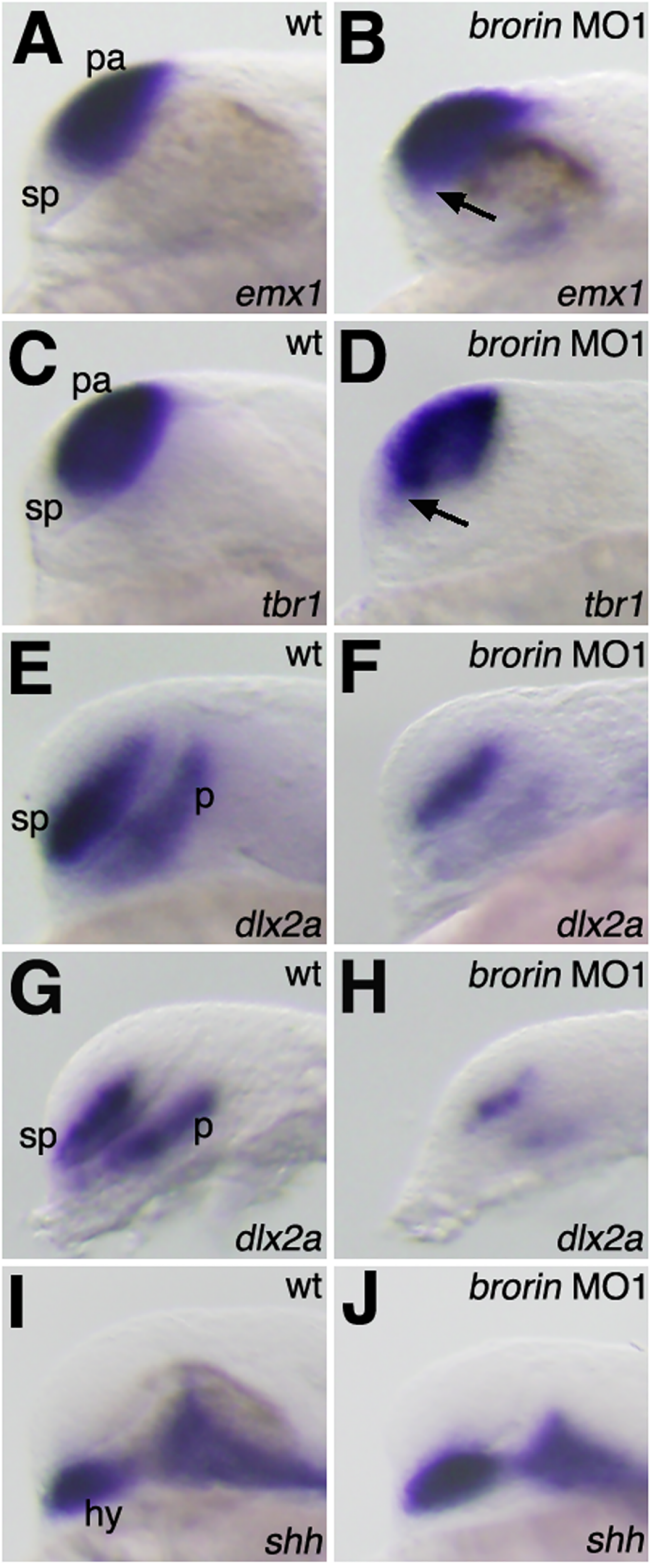
Telencephalic and diencephalic gene expression in *brorin* morphants. (A-D) The expression of *emx1* (A, B) and *tbr1* (C, D) in wild-type embryos (A, C) and *brorin* morphants (B, D) at 24 hpf. Arrows in panels B and D indicate the ectopic expression of *emx1* or *tbr1* in the subpallial domain of the telencephalon. (E-H) The expression of *dlx2a* in wild-type embryos (E, G) and *brorin* morphants (F, H) at 8 (G, H) and 24 (E, F) hpf. (I, J) The expression of *shh* in wild-type embryos (I) and *brorin* morphants (J) at 24 hpf. hy, hypothalamus; p, prethalamus; pa, pallial telencephalon; sp, subpallial telencephalon.

The pattern of *brorin* expression in the embryonic zebrafish brain and phenotypic changes in *brorin* MO-injected embryos suggest that *brorin* is involved in the formation of the forebrain. Bmp signaling participates in forebrain patterning [3–7]. Therefore, in order to investigate the involvement of *brorin* in forebrain regionalization, we examined the expression of telencephalon marker genes in *brorin* morphants. In wild-type embryos, the expression of *emx1* and *tbr1* (pallial telencephalon marker genes) was not detected in the subpallial region of the telencephalon at 24 hpf. In *brorin* morphants, the ectopic expression of *emx1* and *tbr1* was detected in the subpallial domain of the telencephalon (MO1, *n* = 22/22 and *n* = 14/14, respectively) (Fig 6A–6D). In contrast, the expression of *dlx2a*, which is normally detected in the ventral telencephalon, was reduced in *brorin* morphants at 24 hpf (MO1, *n* = 22/23 and MO2, *n* = 9/12) (Fig 6E and 6F and data not shown). Furthermore, a reduction in *dlx2a* expression was observed in the ventral telencephalon of *brorin* morphants at 18 hpf (MO1, *n* = 15/15) (Fig 6G and 6H). These results indicate that *brorin* is required for the development of the subpallial telencephalon. Furthermore, we investigated whether the knockdown of *brorin* had an effect on diencephalic specification at 24 hpf. The expression of *dlx2a* is normally detected in the prethalamus, but was weakly expressed in *brorin* morphants (MO1, *n* = 22/23 and MO2, *n* = 9/12) (Fig 6E and 6F and data not shown). A reduction in *dlx2a* expression in the prethalamus was also observed in *brorin* morphants at 18 hpf (MO1, *n* = 15/15) (Fig 6G and 6H). However, the expression of *shh* in the floor plate and hypothalamus was unaffected in *brorin* morphants (MO1, *n* = 19/19) (Fig 6I and 6J). These results indicate that *brorin* is required for the complete initiation of *dlx2a* expression in the forebrain.

### Effects of *brorin* knockdown on development of GABAergic neurons, oligodendrocytes, and astroglia

**Fig 7:**
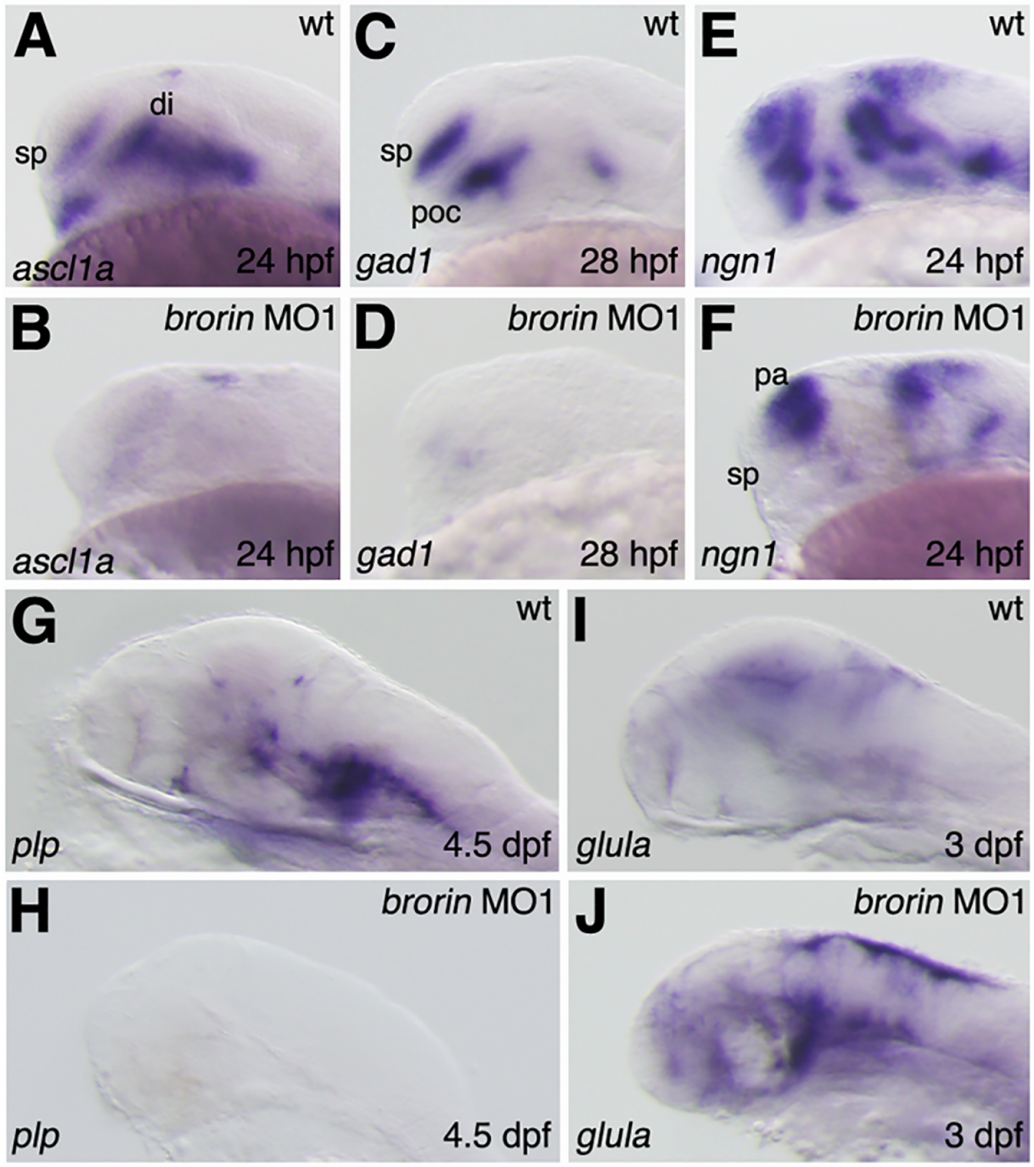
Specification of GABAergic interneurons and oligodendrocytes, and astroglial development in *brorin* morphants. The expression of *ascl1a* (A, B), *gad1* (C, D), *ngn1* (E, F), *plp* (G, H), and *glula* (I, J) in wild-type embryos (A, C, E, G, I) and *brorin* morphants (B, D, F, H, J) is displayed at the indicated stages. di, diencephalon; pa, pallial telencephalon; poc, postoptic commissure; sp, subpallial telencephalon.

GABAergic interneurons and oligodendrocytes originate from the subpallial telencephalon and ventral thalamus of the forebrain, and *Dlx2* participates in the specification of GABAergic interneurons and oligodendrocytes [47–51]. The reduced expression of *dlx2a* in *brorin* morphants suggests an effect on the specification of GABAergic interneurons and oligodendrocytes in the ventral forebrain. In the forebrain, *achaete-scute complex* (*ascl*) *1a* is expressed by GABAergic interneurons and their precursors [52]. *gad1*, which encodes glutamic acid decarboxylase, is also specifically expressed by GABAergic interneurons [28]. In order to examine whether the knockdown of *brorin* affects the differentiation of forebrain GABAergic interneurons, the expression of *ascl1a* and *gad1* was analyzed in *brorin* morphants at 24 hpf and 28 hpf, respectively. While *ascl1a* expression was normally detected in the ventral telencephalon and diencephalon, it was severely reduced in these regions in *brorin* morphants at 24 hpf (MO1, *n* = 20/20) (Fig 7A and 7B). In addition, *gad1* expression was normal in the subpallial telencephalon and nucleus of the tract of the postoptic commissure (POC), but was markedly reduced in *brorin* morphants at 28 hpf (MO1, *n* = 17/17 and MO2, *n* = 7/7) (Figs 7C and 7D and S1A Fig). The reduction in *gad1* expression in *brorin* morphants was prevented by the co-injection of *brorin* RNA with *brorin* MO1 (*n* = 15/15) (S1B Fig). These results demonstrate that the specification of forebrain GABAergic interneurons is suppressed in *brorin* morphants. We then investigated whether the knockdown of *brorin* affected neuronal differentiation in the pallial telencephalon at 24 hpf. The expression of *ngn1*, which is a basic helix-loop helix (bHLH) proneural gene, was analyzed in *brorin* morphants. In wild-type embryos, a narrow region that did not express *ngn1* was observed in the pallial telencephalon, while the expression of *ngn1* was up-regulated and the region that did not express *ngn1* was undetectable in the pallial telencephalon of *brorin* morphants (MO1, *n* = 16/16) (Fig 7E and 7F). However, the expression of *ngn1* was not detected in the subpallial telencephalon of *brorin* morphants, in contrast to other pallial telencephalon markers (MO1, *n* = 16/16) (Fig 7E and 7F). These results suggest that neuronal differentiation in the pallial telencephalon and subpallial telencephalon is affected in *brorin* morphants. In addition to the pallial telencephalon, *ngn1* is normally expressed in the ventral diencephalon; however, its expression in the ventral diencephalon was reduced in *brorin* morphants (MO1, *n* = 15/16) (Fig 7E and 7F). This result indicates that neuronal differentiation in the ventral diencephalon is affected in *brorin* morphants and is consistent with the above results.

We also examined the involvement of *brorin* in the development of oligodendrocytes. The expression of *PLP (proteolipid protein)/DM20*, a marker of oligodendrocyte differentiation, was analyzed in *brorin* morphants. *plp* expression was strongly reduced in the brains of *brorin* morphants at 4.5 dpf (MO1, *n* = 16/16 and MO2, *n* = 7/7) (Fig 7G and 7H and S1C Fig). On the other hand, the co-injection of *brorin* RNA with *brorin* MO1 prevented the reduction in *plp* expression caused by *brorin* MO1 (*n* = 8/11) (S1D Fig). This result indicates that the development of oligodendrocytes in the brain is suppressed by the knockdown of *brorin*. We also examined whether the knockdown of *brorin* affected the development of astroglia. The expression of *glula* (glutamine synthetase) was analyzed in *brorin* morphants because *Glul* (*Glns*) is predominantly expressed in astrocyte precursors and astrocytes [30]. *glula* expression was markedly up-regulated in the brains of *brorin* morphants at 3 dpf (MO1, *n* = 9/10 and MO2, *n* = 9/11) (Figs 7I and 7J and S1E Fig). The co-injection of *brorin* RNA with *brorin* MO1 suppressed the increased expression of *glula* caused by *brorin* MO1 (*n* = 11/12) (S1F Fig). These results demonstrate that astroglial development is facilitated by the knockdown of *brorin*.

### Effects of *brorin* knockdown on axon guidance

**Fig 8:**
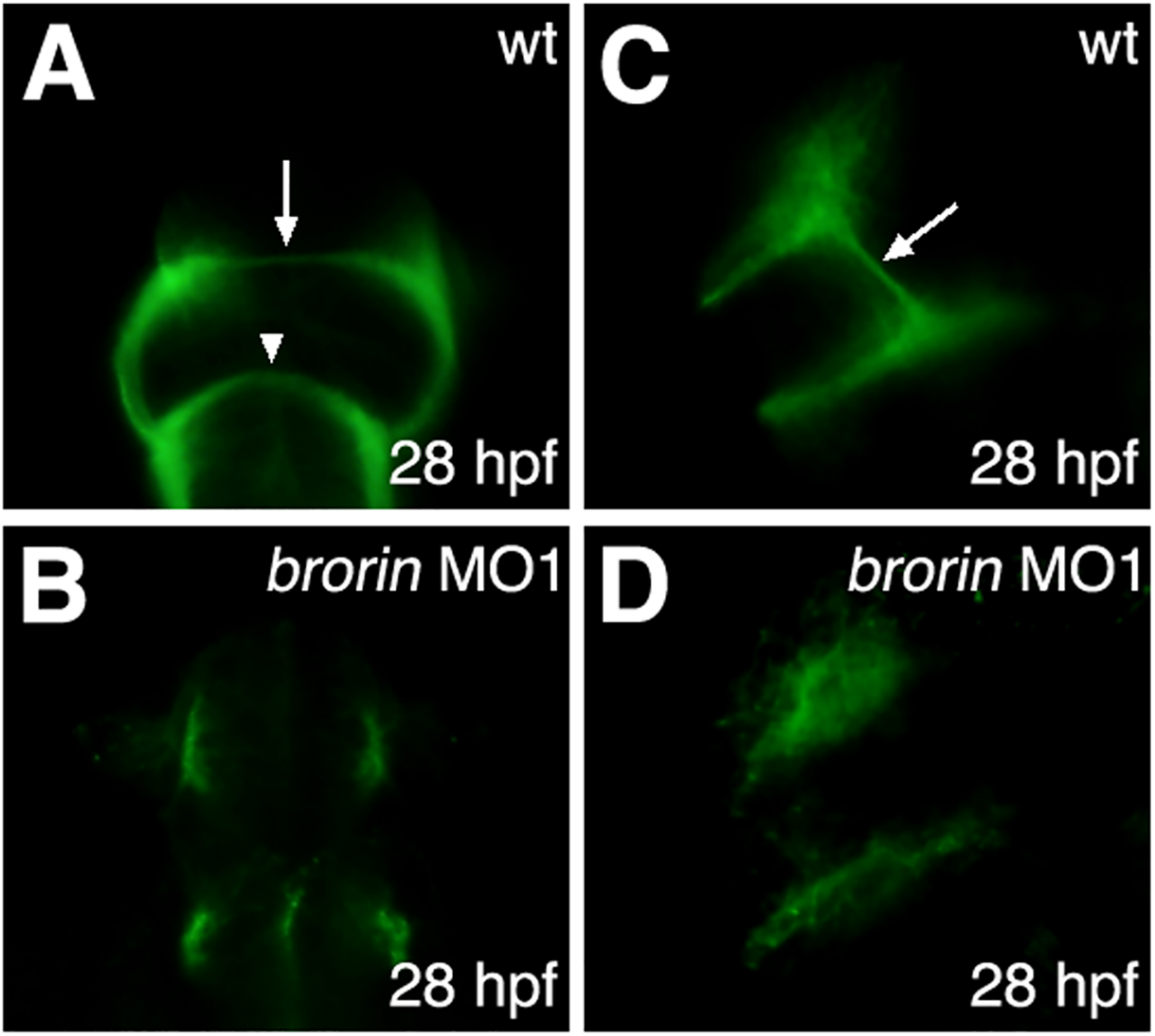
Defects in axon guidance in *brorin* morphants. Fluorescent immunolabeling of axons (*α*AT) in wild-type embryos (A, C) and *brorin* morphants (B, D) at 28 hpf. The arrow and arrowhead in panel A indicate the AC and POC, respectively. The arrow in panel C indicates the SOT. A and B are frontal views; C and D are lateral views, with the anterior to the left.

We investigated the involvement of *brorin* in the formation of commissures because its expression was confined to the region adjacent to the anterior commissure (AC) and tract of the POC in the forebrain by 36 hpf. We used an antibody against acetylated α-tubulin to examine the formation of forebrain commissures in *brorin* morphants. In wild-type embryos, neurons of the dorsorostral cluster in the telencephalon projected contralaterally to form the AC at 28 hpf (Fig 8A) [53,54]. Furthermore, they extended axons ventrally towards the ventrorostral cluster forming the supraoptic tract (SOT) (Fig 8C) [53,54]. Neurons of the ventrorostral cluster in the diencephalon projected contralaterally to form the POC (Fig 8A) [55]. Although axons from the nucleus of the tract of the AC in the telencephalon and axons from the nucleus of the tract of the POC in the diencephalon were present in *brorin* morphants, these axons did not extend across the midline and forebrain commissures were not formed (MO1, *n* = 23/23) (Fig 8B). In addition, the SOT was not detected in *brorin* morphants (MO1, *n* = 23/23) (Fig 8D). These results indicate that the knockdown of *brorin* affects axon guidance in the forebrain.

**Fig 9:**
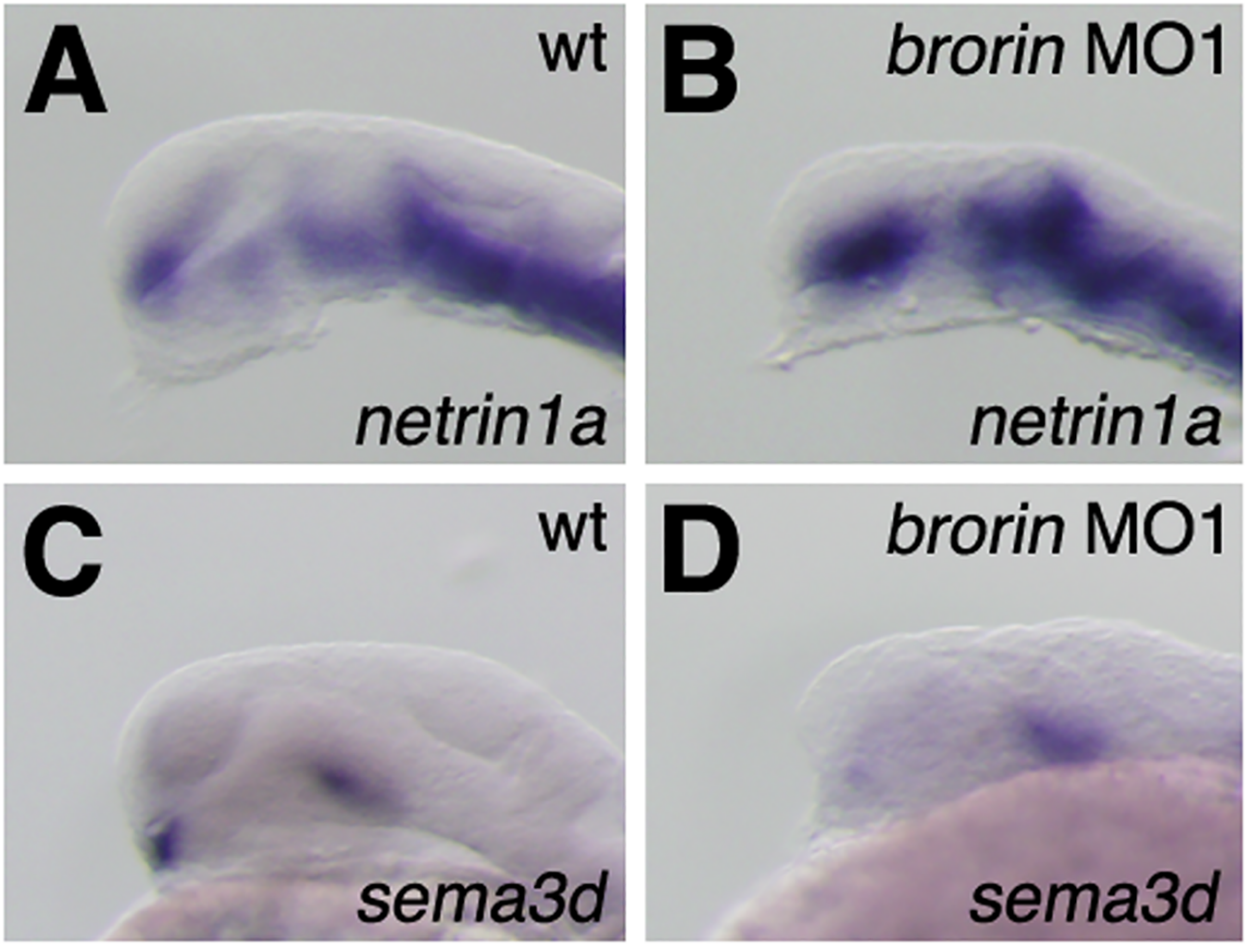
Expression of axon growth substrates in *brorin* morphants. Expression of *netrin1a* (A, B) and *sema3d* (C, D) in wild-type embryos (A, C) and *brorin* morphants (B, D) at 24 hpf.

In order to clarify whether *brorin* is involved in establishing the commissural axon growth substrate, we analyzed the expression of axon guidance molecules (*netrin1a* and *sema3d*) in *brorin* morphants. In zebrafish, *netrin1a* is normally absent from the diencephalon, in which the POC forms, whereas *netrin1a* expression was up-regulated in the telencephalon and expanded across the optic recess into the ventral thalamus in *brorin* morphants (MO1, *n* = 16/17 and MO2, *n* = 8/9) (Figs 9A and 9B and S1G Fig). The co-injection of *brorin* RNA with *brorin* MO1 suppressed the increased expression of *netrin1a* caused by *brorin* MO1 (*n* = 10/13) (S1H Fig). In contrast, *sema3d* expression was normally detected at the midline of the diencephalon immediately ventral to the POC, but was reduced in the diencephalon in *brorin* morphants (MO1, *n* = 15/15) (Fig 9C and 9D).

## Discussion

### *brorin* inhibits Bmp signaling

Bmps play a crucial role in the diverse processes of morphogenesis and development [15, 56], and are subjected to negative or positive regulation by various secreted regulators, including Chordin family members [15]. The Chordin family of proteins possesses three to eighteen cysteine-rich domains, each consisting of 10 cysteine residues [18,57,58]. Mouse Brorin is a neural-specific secreted antagonist of Bmp signaling with two cysteine-rich domains in its core region, and the cysteine residues in these domains are located at similar positions to those in other Chordin family members [16]. Among the Chordin family members, these cysteine-rich domains are the most similar to those of Crossveinless-2, which functions as a Bmp antagonist and pro-Bmp factor [16,58]. However, the amino acid sequence of mouse Brorin does not share structural similarities with other members of the Chordin family, and Brorin is a unique member of this family. The core region of zebrafish Brorin also has two cysteine-rich domains. The amino acid sequence of the 127-amino acid amino-terminal region of zebrafish Brorin was less similar to that of mouse Brorin, although the other regions of zebrafish Brorin (182 amino acids) were highly similar to mouse Brorin. We concluded that Brorin is a zebrafish ortholog of mouse Brorin based on the conservation of the intron-exon organization and syntenic relationship. Our results suggested that the functional region of Brorin was located in the core region containing the two cysteine-rich domains.

Exogenous Brorin has been shown to inhibit the phosphorylation of Smad by Bmp2 and Bmp6 as well as osteoblastic differentiation *in vitro* [16]. In zebrafish, Bmps are essential for ventralization of the embryo and the inhibition of Bmp signaling results in embryos that are devoid of the non-axial region of the tail [42–46]. We found that the overexpression of *brorin* inhibited the phosphorylation of Smad at the gastrulation stage and led to defects in the tail in zebrafish embryos. Furthermore, we observed the loss of pSmad in the forebrain of *brorin* RNA-injected embryos and an increase in pSmad in the brains of *brorin* morphants at 24 hpf. Embryos injected with *brorin* RNA and *brorin* morphants both exhibited morphological abnormalities in the brain. On the other hand, the Wnt signaling pathway was not promoted by the knockdown of *brorin*, although the inhibition of Wnt signaling leads to the dorsalization of zebrafish embryos and results in a similar phenotype to that caused by the inhibition of Bmp signaling. These results indicate that Brorin inhibits Bmp signaling, but not Wnt signaling, suggesting that it acts as a Bmp antagonist *in vivo*.

### *brorin* is involved in forebrain development

In the telencephalon, *dlx2a* expression was decreased in the ventral region at 24 hpf by the knockdown of *brorin*. In addition, *brorin* morphants exhibited the ectopic expression of markers of the pallial telencephalon in the ventral telencephalon along with the reduced expression of a marker of the subpallial telencephalon. These results indicate that brorin is required for the development of the subpallial telencephalon. Furthermore, the activation of Bmp signaling was observed in the dorsal region of the brain in *brorin* morphants at 24 hpf. Bmp activity from the roof plate was previously shown to be involved in patterning of the dorsal telencephalon [4–7]. These findings suggest that alterations in gene expression in the subpallial telencephalon of *brorin* morphants are due to increases in Bmp activity in the dorsal region of the brain.

Wnt and Fgf are also involved in forebrain patterning and previous studies reported that the cross-regulation of Bmp, Wnt, and Fgf signaling in the early telencephalon is required to pattern the cerebral cortex [4–6]. Excess Fgf8 has no effects on *Bmp4* expression in the cortical hem, whereas an increase in Bmp activity suppresses *Fgf8* expression in the anterior telencephalon and anterior neural ridge [6]. On the other hand, the inhibition of Bmp signaling results in the loss of expression of *Wnt* genes in the cortical hem and excess Fgf8 also suppresses the expression of *Wnt* genes [6,7]. However, *axin2* expression was not increased in the forebrain of *brorin* morphants. Since Bmp antagonists other than *brorin* are also expressed in the forebrain, we speculate that the activation of Bmp signaling caused by the inhibition of *brorin* alone may be insufficient to activate of Wnt signaling. Furthermore, the inhibition of both *fgf3* and *fgf8* has been shown to suppress *tbr1* expression [59]. However, the expansion of *tbr1* expression was detected in the telencephalon of *brorin* morphants. Therefore, patterning of the telencephalon by *brorin* may not be mediated through the Fgf signaling pathway. A direct role for Shh in patterning of the dorsal telencephalon has also been reported [60]. However, the phenotype of *brorin* RNA-injected embryos was not similar to that of *shh* RNA-injected embryos, because the overexpression of *shh* results in abnormalities in the forebrain and eyes, but not in the tail [61,62]. Thus, *brorin* has been suggested to play a role in patterning of the telencephalon by inhibiting Bmp signaling and the inactivation of Bmp signaling by *brorin* is not mediated through Shh.

In the diencephalon, *dlx2a* expression was also decreased in the prethalamus at 18 and 24 hpf by the knockdown of *brorin*, whereas *shh* expression was unaffected in the hypothalamus. These results demonstrate that *brorin* is required for the complete initiation of *dlx2a* expression in the prethalamus. Thus, we expect *brorin* to be involved in patterning of the forebrain through its inhibition of Bmp signaling.

### *brorin* is required for the specification of oligodendrocyte progenitors and GABAergic interneurons and inhibits astrogliogenesis in the forebrain

Previous studies reported that Ngn1 confers neuronal identity on uncommitted precursors and is essential for neurogenesis [63–65]. Brorin was shown to be involved in neuronal differentiation *in vitro* [16]. In *brorin* morphants, the expression of *ngn1* was reduced in the ventral diencephalon, but was up-regulated in the pallial telencephalon. These results suggest that *brorin* modulates neuronal differentiation in the telencephalon and diencephalon. The ectopic expression of *Dlx2* in cortical explants results in the induction of the GABAergic marker *GAD1*, and *Ascl1* is also required for proper GABAergic specification [66,67]. The present study showed that the expression of *dlx2a* and *ascl1a* was reduced in the forebrains of *brorin* morphants. Furthermore, the knockdown of *brorin* resulted in a marked reduction in the expression of *gad1* in the ventral telencephalon and diencephalon. Accordingly, *brorin* appears to play a crucial role in the differentiation of GABAergic interneurons.

*PLP* is expressed in oligodendrocyte progenitor cells [68–70]. The knockdown of *brorin* resulted in a marked reduction in *plp* expression, in addition to the decreased expression of *dlx2a*, in the brain. However, the expression of *glula*, a marker of the astroglial lineage, was increased in the forebrain of *brorin* morphants. These results demonstrated that the knockdown of *brorin* suppresses oligodendrogliogenesis and promotes astrogliogenesis in the forebrain. Thus, *brorin* is required for the specification of oligodendrocyte progenitors and is involved in suppressing the development of astroglia in the forebrain. The knockdown of *brorin* led to the activation of Bmp signaling. The repression of the Bmp pathway is known to be required for oligodendroglial specification during development of the vertebrate brain [10]. Bmp signaling inhibits the specification of oligodendrocytes from neural progenitor cells and promotes the generation of astrocytes [8–10]. Consequently, Bmps promote the differentiation of glial progenitors toward the astroglial lineage. Accordingly, brorin may regulate glial cell differentiation toward an oligodendroglial fate by repressing the Bmp pathway.

### *brorin* is required for the appropriate expression of axon guidance molecules and axon guidance

The AC, POC, and SOT were absent in *brorin* morphants, demonstrating that the loss of *brorin* influences commissure formation. In vertebrates, commissural axon crossing is regulated by a combination of attractive and repulsive cues [71–74]. Netrins attract commissural axon growth cones toward the midline of the central nervous system (CNS), whereas Semaphorins typically repel growth cones [75–77]. In *brorin* morphants, the expression of *sema3d* was lost in the diencephalon, whereas it was unaffected in the midbrain. Furthermore, the expression of *netrin1a* was increased in the telencephalon and expanded across the commissure region in the diencephalon, indicating that *brorin* is required for the proper expression of *netrin1a* and *sema3d* in the forebrain. Accordingly, *brorin* may regulate commissure formation by modulating the expression of axon guidance molecules. However, Brorin itself may be an axon guidance molecule because Bmps have been shown to influence axon guidance in the CNS by acting directly on axons [78].

## Conclusions

*brorin* inhibits Bmp signaling in the zebrafish and is involved in the development of the forebrain, including the specification of GABAergic interneurons and oligodendrocytes as well as the inhibition of astrocyte generation. Furthermore, *brorin* is required for the appropriate expression of axon guidance molecules and commissure formation in the forebrain. These results implicate *brorin* in regionalization, cell-type specification, and axon guidance through the repression of Bmp signaling during zebrafish forebrain development. The amino acid sequence of Brorin is similar to that of Brorin-like/vwc2l, and we previously reported the expression of *brorin-like* and phenotype of *brorin-like* knockdown [17]. In the forebrain, the expression pattern of *brorin* is similar to, but distinct from that of *brorin-like*. At 36 hpf, *brorin* expression was detected in the ventral telencephalon, while *brorin-like* was not expressed in the telencephalon [17]. On the other hand, *brorin* and *brorin-like* were both expressed in the prethalamic/alar hypothalamic region [17]. In addition, the phenotype of *brorin* knockdown is similar to, but distinct from that of *brorin-like*. Therefore, we conclude that *brorin* has unique roles in the development of the forebrain. However, *brorin* and *brorin-like* may in part function redundantly during forebrain development. This will be addressed in a future study.

## Supporting information

S1 Fig Effects of *brorin* knockdown on the specification of GABAergic interneurons, oligodendrocytes, and astrocytes, and axon guidance. The expression of *gad1* (A, B), *plp* (C, D), *glula* (E, F), and *netrin1a* (G, H) in *brorin* MO2-injected (A, C, E, G) and *brorin* MO1- and *brorin* RNA-injected (B, D, F, H) embryos is displayed at the indicated stages.
(TIF)

## References

[1] Hauptmann G, Söll I, Gerster T. The early embryonic zebrafish forebrain is subdivided into molecularly distinct transverse and longitudinal domains. Brain Res Bull. 2002;57(3–4): 371–375. 11922991 10.1016/s0361-9230(01)00691-8

[2] Lauter G, Söll I, Hauptmann G. Molecular characterization of prosomeric and intraprosomeric subdivisions of the embryonic zebrafish diencephalon. J Comp Neurol. 2013;521(5): 1093–1118. doi: 10.1002/cne.23221 22949352 10.1002/cne.23221

[3] Wilson SW, Rubenstein JLR. Induction and dorsoventral patterning of the telencephalon. Neuron. 2000;28(3): 641–651. 11163256 10.1016/s0896-6273(00)00171-9

[4] Monuki ES, Porter FD, Wslsh CA. Patterning of the dorsal telencephalon and cerebral cortex by a roof plate-Lhx2 pathway. Neuron. 2001;32(4): 591–604. 11719201 10.1016/s0896-6273(01)00504-9

[5] Theil T, Aydin S, Koch S, Grotewold L, Rüther U. Wnt and Bmp signalling cooperatively regulate graded Emx2 expression in the dorsal telencephalon. Development. 2002;129(13): 3045–3054. 12070081 10.1242/dev.129.13.3045

[6] Shimogori T, Banuchi V, Ng HY, Strauss JB, Grove EA. Embryonic signaling centers expressing BMP, WNT and FGF proteins interact to pattern the cerebral cortex. Development. 2004;131(22): 5639–5647. doi: 10.1242/dev.01428 15509764 10.1242/dev.01428

[7] Cheng X, Hsu CM, Currle DS, Hu JS, Barkovich AJ, et al Central roles of the roof plate in telencephalic development and holoprosencephaly. J Neurosci. 2006;26(29): 7640–7649. doi: 10.1523/JNEUROSCI.0714-06.2006 16855091 10.1523/JNEUROSCI.0714-06.2006 PMC6674267

[8] Nakashima K, Takizawa T, Ochiai W, Yanagisawa M, Hisatsune T, et al BMP2-mediated alteration in the developmental pathway of fetal mouse brain cells from neurogenesis to astrocytogenesis. Proc Natl Acad Sci U S A. 2001;98(10): 5868–5873. doi: 10.1073/pnas.101109698 11331769 10.1073/pnas.101109698 PMC33305

[9] Yanagisawa M, Takizawa T, Ochiai W, Uemura A, Nakashima K, et al Fate alteration of neuroepithelial cells from neurogenesis to astrocytogenesis by bone morphogenetic proteins. Neurosci Res. 2001;41(4): 391–396. 11755226 10.1016/s0168-0102(01)00297-8

[10] See J, Mamontov P, Ahn K, Wine-Lee L, Crenshaw EB 3rd, et al BMP signaling mutant mice exhibit glial cell maturation defects. Mol Cell Neurosci. 2007;35(1): 171–182. doi: 10.1016/j.mcn.2007.02.012 17391983 10.1016/j.mcn.2007.02.012 PMC1950488

[11] Briscoe J, Chen Y, Jessell TM, Struhl G. A hedgehog-insensitive from of patched provides evidence for direct long-range morphogen activity of sonic hedgehog in the neural tube. Mol Cell. 2001;7(6): 1279–1291. 11430830 10.1016/s1097-2765(01)00271-4

[12] Briscoe J, Chen Y, Jessell TM, Struhl G. A hedgehog-insensitive from of patched provides evidence for direct long-range morphogen activity of sonic hedgehog in the neural tube. Mol Cell. 2001;7(6): 1279–1291. 11430830 10.1016/s1097-2765(01)00271-4

[13] Varga ZM, Amores A, Lewis KE, Yan YL, Postlethwait JH. Zebrafish *smoothene*d functions in ventral neural tube specification and axon tract formation. Development. 2001;128(18): 3497–3509. 11566855 10.1242/dev.128.18.3497

[14] Kingsley DM. The TGF-beta superfamily: new members, new receptors, and new genetic tests of function in different organisms. Genes Dev. 1994;8(2): 133–146. 8299934 10.1101/gad.8.2.133

[15] Balemans W, Van Hul W. Extracellular regulation of BMP signaling in vertebrates: a cocktail of modulators. Dev Biol. 2002;250(2): 231–250. 12376100

[16] Koike N, Kassai Y, Kouta Y, Miwa H, Konishi M, et al Brorin, a novel secreted bone morphogenetic protein antagonist, promotes neurogenesis in mouse neural precursor cells. J Biol Chem. 2007;282(21): 15843–15850. doi: 10.1074/jbc.M701570200 17400546 10.1074/jbc.M701570200

[17] Miwa H, Miyake A, Kouta Y, Shimada A, Yamashita Y, et al A novel neural-specific BMP antagonist, Brorin-like, of the Chordin family. FEBS Lett. 2009;583(22): 3643–3648. doi: 10.1016/j.febslet.2009.10.044 19852960 10.1016/j.febslet.2009.10.044

[18] Garcia Abreu J, Coffinier C, Larraín J, Oelgeschläger M, De Robertis EM. Chordin-like CR domains and the regulation of evolutionarily conserved extracellular signaling systems. Gene. 2002;287(1–2): 39–47. 11992721 10.1016/s0378-1119(01)00827-7

[19] Kimmel CB, Ballard WW, Kimmel SR, Ullmann B, Schilling TF. Stages of embryonic development of the zebrafish. Dev Dyn. 1995;203(3): 253–310. doi: 10.1002/aja.1002030302 8589427 10.1002/aja.1002030302

[20] Miyake A, Nakayama Y, Konishi M, Itoh N. *Fgf19* regulated by Hh signaling is required for zebrafish forebrain development. Dev Biol. 2005;288(1): 259–275. doi: 10.1016/j.ydbio.2005.09.042 16256099 10.1016/j.ydbio.2005.09.042

[21] Hauptmann G, Gerster T. Two-color whole-mount *in situ* hybridization to vertebrate and *Drosophila* embryos. Trends Genet. 1994;10(8): 266 7940754 10.1016/0168-9525(90)90008-t

[22] Morita T, Nitta H, Kiyama Y, Mori H, Mishina M. Differential expression of two zebrafish *emx* homeoprotein mRNAs in the developing brain. Neurosci Lett. 1995;198(2): 131–134. 8592638 10.1016/0304-3940(95)11988-9

[23] Mione M, Shanmugalingam S, Kimelman D, Griffin K. Overlapping expression of zebrafish T-brain-1 and eomesodermin during forebrain development. Mech Dev. 2001;100(1): 93–97. 11118891 10.1016/s0925-4773(00)00501-3

[24] Akimenko MA, Ekker M, Wegner J, Lin W, Westerfield M. Combinatorial expression of three zebrafish genes related to *distal-less*: part of a homeobox gene code for the head. J Neurosci. 1994;14(6): 3475–3486. 7911517 10.1523/JNEUROSCI.14-06-03475.1994 PMC6576961

[25] Krauss S, Concordet JP, Ingham PW. A functionally conserved homolog of the Drosophila segment polarity gene hh is expressed in tissues with polarizing activity in zebrafish embryos. Cell. 1993;75(7): 1431–1444. 8269519 10.1016/0092-8674(93)90628-4

[26] Korzh V, Sleptsova I, Liao J, He J, Gong Z. Expression of zebrafish bHLH genes *ngn1* and *nrd* defines distinct stages of neural differentiation. Dev Dyn. 1998;213(1): 92–104. doi: 10.1002/(SICI)1097-0177(199809)213:1<92::AID-AJA9>3.0.CO;2-T 9733104 10.1002/(SICI)1097-0177(199809)213:1<92::AID-AJA9>3.0.CO;2-T

[27] Allende ML, Weinberg ES. The expression pattern of two zebrafish *achaete-scute* homolog (*ash*) genes is altered in the embryonic brain of the *cyclops* mutant. Dev Biol. 1994;166(2): 509–530. doi: 10.1006/dbio.1994.1334 7813774 10.1006/dbio.1994.1334

[28] Martin SC, Heinrich G, Sandell JH. Sequence and expression of glutamic acid decarboxylase isoforms in the developing zebrafish. J Comp Neurol. 1998;396(2): 253–266. 9634146

[29] Park H-C, Mehta A, Richardson JS, Appel B. *olig2* is required for zebrafish primary motor neuron and oligodendrocyte development. Dev Biol. 2002;248(2): 356–368. 12167410 10.1006/dbio.2002.0738

[30] Esain V, Postlethwait JH, Charnay P, Ghislain J. FGF-receptor signalling controls neural cell diversity in the zebrafish hindbrain by regulating *olig2* and *sox9*. Development. 2010;137(1): 33–42. doi: 10.1242/dev.038026 20023158 10.1242/dev.038026 PMC2796930

[31] Lauderdale JD, Davis NM, Kuwada JY. Axon tracts correlate with *netrin-1a* expression in the zebrafish embryo. Mol Cell Neurosci. 1997;9(4): 293–313. doi: 10.1006/mcne.1997.0624 9268507 10.1006/mcne.1997.0624

[32] Seth A, Culverwell J, Walkowicz M, Toro S, Rick JM, et al *belladonna*/(*lhx2*) is required for neural patterning and midline axon guidance in the zebrafish forebrain. Development. 2006;133(4): 725–735. doi: 10.1242/dev.02244 16436624 10.1242/dev.02244

[33] Turner DL, Weintraub H. Expression of achaete-scute homolog 3 in *Xenopus* embryos converts ectodermal cells to a neural fate. Genes Dev. 1994;8(12): 1434–1447. 7926743 10.1101/gad.8.12.1434

[34] Nasevicius A, Ekker SC. Effective targeted gene ‘knockdown’ in zebrafish. Nat Genet. 2000;26(2): 216–220. doi: 10.1038/79951 11017081 10.1038/79951

[35] Miyake A, Nihno S, Murakoshi Y, Satsuka A, Nakayama Y, et al Neucrin, a novel secreted antagonist of canonical Wnt signaling, plays roles in developing neural tissues in zebrafish. Mech Dev. 2012;128(11–12): 577–590. doi: 10.1016/j.mod.2012.01.001 22265871 10.1016/j.mod.2012.01.001

[36] Miyake A, Itoh N. Fgf22 regulated by Fgf3/Fgf8 signaling is required for zebrafish midbrain development. Biol Open. 2013;2(5): 515–524. doi: 10.1242/bio.20134226 23789101 10.1242/bio.20134226 PMC3654271

[37] Miyake A, Chitose T, Kamei E, Murakami A, Nakayama Y, et al *Fgf16* is required for specification of GABAergic neurons and oligodendrocytes in the zebrafish forebrain. PLoS One. 2014;9(10): e110836 doi: 10.1371/journal.pone.0110836 25357195 10.1371/journal.pone.0110836 PMC4214708

[38] French CR, Erickson T, French DV, Pilgrim DB, Waskiewicz AJ. Gdf6a is required for the initiation of dorsal-ventral retinal patterning and lens development. Dev Biol. 2009;333(1): 37–47. doi: 10.1016/j.ydbio.2009.06.018 19545559 10.1016/j.ydbio.2009.06.018

[39] Maurya AK, Tan H, Souren M, Wang X, Wittbrodt J, et al Integration of Hedgehog and BMP signalling by the *engrailed2a* gene in the zebrafish myotome. Development. 2011;138(4): 755–765. doi: 10.1242/dev.062521 21266411 10.1242/dev.062521

[40] Wilson SW, Ross LS, Parrett T, Easter SSJ. The development of a simple scaffold of axon tracts in the brain of the embryonic zebrafish, Brachydanio rerio. Development. 1990;108(1): 121–145. 2351059 10.1242/dev.108.1.121

[41] Nohe A, Keating E, Knaus P, Petersen NO. Signal transduction of bone morphogenetic protein receptors. Cell Signal. 2004;16(3): 291–299. 14687659 10.1016/j.cellsig.2003.08.011

[42] Kishimoto Y, Lee KH, Zon L, Hammerschmidt M, Schulte-Merker S. The molecular nature of zebrafish swirl: BMP2 function is essential during early dorsoventral patterning. Development. 1997;124(22): 4457–4466. 9409664 10.1242/dev.124.22.4457

[43] Dick A, Hild M, Bauer H, Imai Y, Maifeld H, et al Essential role of Bmp7 (snailhouse) and its prodomain in dorsoventral patterning of the zebrafish embryo. Development. 2000;127(2): 343–354. 10603351 10.1242/dev.127.2.343

[44] Schmid B, Fürthauer M, Connors SA, Trout J, Thisse B, et al Equivalent genetic roles for bmp7/snailhouse and bmp2b/swirl in dorsoventral pattern formation. Development. 2000;127(5): 957–967. 10662635 10.1242/dev.127.5.957

[45] Hild M, Dick A, Rauch GJ, Meier A, Bouwmeester T, et al The smad5 mutation somitabun blocks Bmp2b signaling during early dorsoventral patterning of the zebrafish embryo. Development. 1999;126(10): 2149–2159. 10207140 10.1242/dev.126.10.2149

[46] Agathon A, Thisse C, Thisse B. The molecular nature of the zebrafish tail organizer. Nature. 2003;424(6947): 448–452. doi: 10.1038/nature01822 12879074 10.1038/nature01822

[47] Corbin JG, Nery S, Fishell G. Telencephalic cells take a tangent: non-radial migration in the mammalian forebrain. Nat Neurosci. 2001;4: 1177–1182. doi: 10.1038/nn749 11687827 10.1038/nn749

[48] Jones EG. Dichronous appearance and unusual origins of GABA neurons during development of the mammalian thalamus. Thalamus Relat Syst. 2001;1: 283–288.

[49] Marin O, Rubenstein JLR. A long, remarkable journey: tangential migration in the telencephalon. Nat Rev Neurosci. 2001;2(11): 780–790. doi: 10.1038/35097509 11715055 10.1038/35097509

[50] Hayes SG, Murray KD, Jones EG. Two epochs in the development of gamma-aminobutyric acidergic neurons in the ferret thalamus. J Comp Neurol. 2003;463(1): 45–65. doi: 10.1002/cne.10749 12811802 10.1002/cne.10749

[51] Bertrand N, Castro DS, Guillemot F. Proneural genes and the specification of neural cell types. Nat Rev Neurosci. 2002;3(7): 517–530. doi: 10.1038/nrn874 12094208 10.1038/nrn874

[52] Scholpp S, Delogu A, Gilthorpe J, Peukert D, Schindler S, et al Her6 regulates the neurogenetic gradient and neuronal identity in the thalamus. Proc Natl Acad Sci U S A. 2009;106(47): 19895–19900. doi: 10.1073/pnas.0910894106 19903880 10.1073/pnas.0910894106 PMC2775703

[53] Chitnis AB, Kuwada JY. Axonogenesis in the brain of zebrafish embryos. J Neurosci. 1990;10(6): 1892–1905. 2355256 10.1523/JNEUROSCI.10-06-01892.1990 PMC6570297

[54] Wilson SW, Ross LS, Parrett T, Easter SS Jr. The development of a simple scaffold of axon tracts in the brain of the embryonic zebrafish, Brachydanio rerio. Development. 1990;108(1): 121–145. 2351059 10.1242/dev.108.1.121

[55] Bak M, Fraser SE. Axon fasciculation and differences in midline kinetics between pioneer and follower axons within commissural fascicles. Development. 2003;130(20): 4999–5008. doi: 10.1242/dev.00713 12952902 10.1242/dev.00713

[56] von Bubnoff A, Cho KW. Intracellular BMP signaling regulation in vertebrates: pathway or network? Dev Biol. 2001;239(1): 1–14. doi: 10.1006/dbio.2001.0388 11784015 10.1006/dbio.2001.0388

[57] Ueki T, Tanaka M, Yamashita K, Mikawa S, Qiu Z, et al A novel secretory factor, Neurogenesin-1, provides neurogenic environmental cues for neural stem cells in the adult hippocampus. J Neurosci. 2003;23(37): 11732–11740. 14684875 10.1523/JNEUROSCI.23-37-11732.2003 PMC6740945

[58] Coffinier C, Ketpura N, Tran U, Geissert D, De Robertis EM. Mouse *Crossveinless-2* is the vertebrate homolog of a *Drosophila* extracellular regulator of BMP signaling. Mech Dev. 2002;119: S179–184. 14516682 10.1016/s0925-4773(03)00113-8 PMC3039546

[59] Walshe J, Mason I. Unique and combinatorial functions of Fgf3 and Fgf8 during zebrafish forebrain development. Development. 2003;130(18): 4337–4349. 12900450 10.1242/dev.00660

[60] Himmelstein DS, Bi C, Clark BS, Bai B, Kohtz JD. Balanced Shh signaling is required for proper formation and maintenance of dorsal telencephalic midline structures. BMC Dev Biol. 2010;10(118): 693–702. 10.1186/1471-213X-10-118 PMC3018372 21114856

[61] Barth KA, Wilson SW. Expression of zebrafish nk2.2 is influenced by sonic hedgehog/vertebrate hedgehog-1 and demarcates a zone of neuronal differentiation in the embryonic forebrain. Development. 1995;121(6): 1755–1768. 7600991 10.1242/dev.121.6.1755

[62] Macdonald R, Barth KA, Xu Q, Holder N, Mikkola I, Wilson SW. Midline signalling is required for Pax gene regulation and patterning of the eyes. Development. 1995;121(10): 3267–3278. 7588061 10.1242/dev.121.10.3267

[63] Farah MH, Olson JM, Sucic HB, Hume RI, Tapscott SJ, et al Generation of neurons by transient expression of neural bHLH proteins in mammalian cells. Development. 2000;127(4): 693–702. 10648228 10.1242/dev.127.4.693

[64] Nieto M, Schuurmans C, Britz O, Guillemot F. Neural bHLH genes control the neuronal versus glial fate decision in cortical progenitors. Neuron. 2001;29(2): 401–413. 11239431 10.1016/s0896-6273(01)00214-8

[65] Sun Y, Nadal-Vicens M, Misono S, Lin MZ, Zubiaga A, et al Neurogenin promotes neurogenesis and inhibits glial differentiation by independent mechanisms. Cell. 2001;104(3): 365–376. 11239394 10.1016/s0092-8674(01)00224-0

[66] Schuurmans C, Guillemot F. Molecular mechanisms underlying cell fate specification in the developing telencephalon. Curr Opin Neurobiol. 2002;12(1): 26–34. 11861161 10.1016/s0959-4388(02)00286-6

[67] Fode C, Ma Q, Casarosa S, Ang SL, Anderson DJ, et al A role for neural determination genes in specifying the dorsoventral identity of telencephalic neurons. Genes Dev. 2000;14(1): 67–80. 10640277 PMC316337

[68] Timsit S, Martinez S, Allinquant B, Peryon F, Puelles L, et al Oligodentrocytes originate in a restricted zone of the embryonic ventral neural tube defined by DM-20 mRNA expression. J Neurosci. 1995;15(2): 1012–1024. 7869079 10.1523/JNEUROSCI.15-02-01012.1995 PMC6577809

[69] Dickinson PJ, Fanarraga ML, Griffiths IR, Barrie JM, Kyriakides E, et al Oligodendrocyte progenitors in the embryonic spinal cord express DM-20. Neuropathol Appl Neurobiol. 1996;22(3): 188–198. 8804020

[70] Peyron F, Timsit S, Tohmas JL, Kagawa T, Ikenaka K, et al In situ expression of PLP/DM-20, MBP, and CNP during embryonic and postnatal development of jimpy mutant and of transgenic mice overexpressing PLP. J Neurosci Res. 1997;50(2): 190–201. doi: 10.1002/(SICI)1097-4547(19971015)50:2<190::AID-JNR8>3.0.CO;2-A 9373029 10.1002/(SICI)1097-4547(19971015)50:2<190::AID-JNR8>3.0.CO;2-A

[71] Goodman CS. Mechanisms and molecules that control growth cone guidance. Annu Rev Neurosci. 1996;19: 341–377. doi: 10.1146/annurev.ne.19.030196.002013 8833447 10.1146/annurev.ne.19.030196.002013

[72] Dickson BJ. Molecular mechanisms of axon guidance. Science. 2002;298(5600): 1959–1964. doi: 10.1126/science.1072165 12471249 10.1126/science.1072165

[73] Grunwald IC, Klein R. Axon guidance: receptor complexes and signaling mechanisms. Curr Opin Neurobiol. 2002;12(3): 250–259. 12049930 10.1016/s0959-4388(02)00323-9

[74] Steward O. Translating axon guidance cues. Cell. 2002;110(5): 537–540. 12230970 10.1016/s0092-8674(02)00934-0

[75] Harris R, Sabatelli LM, Seeger MA. Guidance cues at the Drosophila CNS midline: identification and characterization of two Drosophila Netrin/UNC-6 homologs. Neuron. 1996;17(2): 217–228. 8780646 10.1016/s0896-6273(00)80154-3

[76] Serafini T, Colamarino SA, Leonardo ED, Wang H, Beddington R, et al Netrin-1 is required for commissural axon guidance in the developing vertebrate nervous system. Cell. 1996;87(6): 1001–1014. 8978605 10.1016/s0092-8674(00)81795-x

[77] Salinas PC. The morphogen sonic hedgehog collaborates with netrin-1 to guide axons in the spinal cord. Trends Neurosci. 2003;26(2): 641–643. 14624844 10.1016/j.tins.2003.09.006

[78] Yamauchi K, Phan KD, Butler SJ. BMP type I receptor complexes have distinct activities mediating cell fate and axon guidance decisions. Development. 2008;135(6): 1119–1128. doi: 10.1242/dev.012989 18272594 10.1242/dev.012989

