# Supplementary figures and images for: Brorin is required for neurogenesis, gliogenesis, and commissural axon guidance in the zebrafish forebrain
Source: PLoS One. 2017 Apr 27;12(4):e0176036. doi: 10.1371/journal.pone.0176036 (PMC5407822; doi:10.1371/journal.pone.0176036)

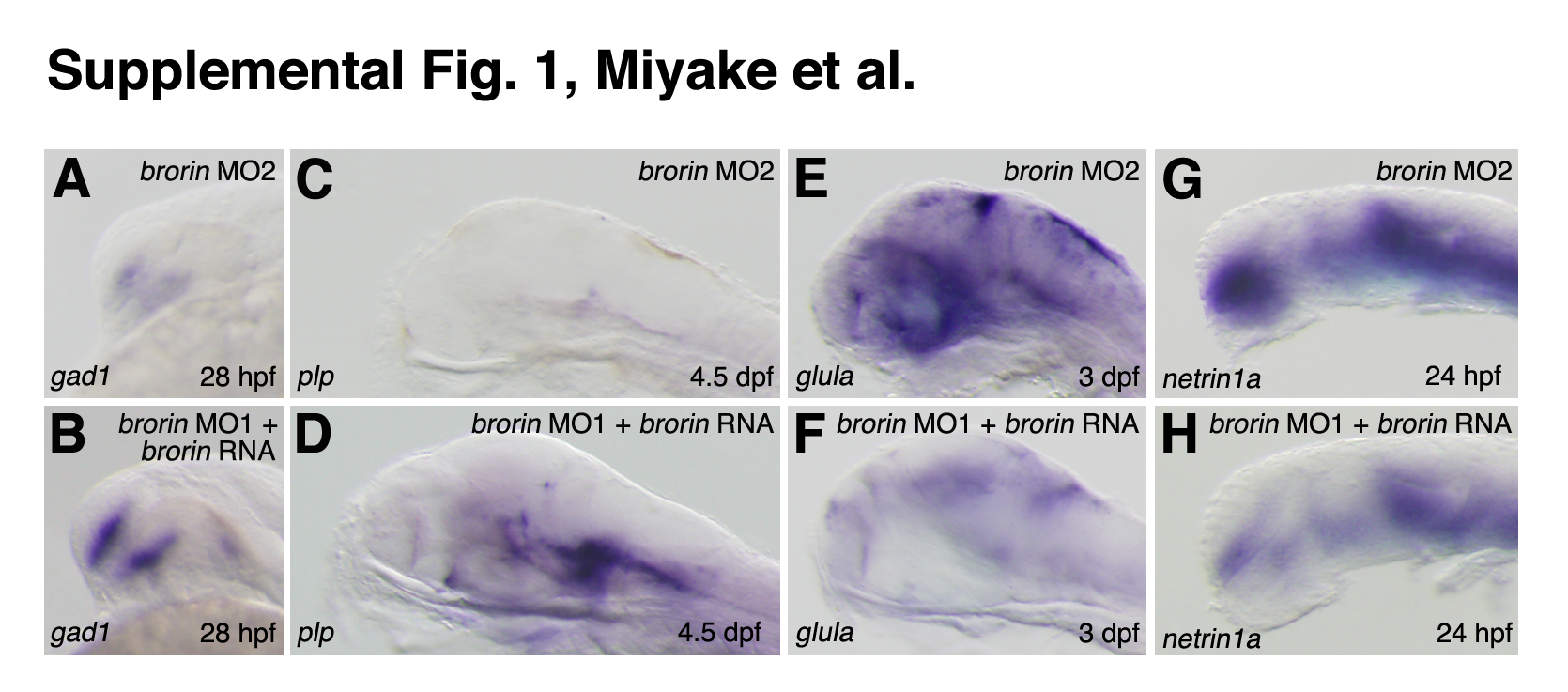

Supplement: S1 Fig — The expression of gad1 (A, B), plp (C, D), glula (E, F), and netrin1a (G, H) in brorin MO2-injected (A, C, E, G) and brorin MO1- and brorin RNA-injected (B, D, F, H) embryos is displayed at the indicated stages. (TIF) [file pone.0176036.s001.tif]
